# Mortality of patients with multiple sclerosis: a cohort study in UK primary care

**DOI:** 10.1007/s00415-014-7370-3

**Published:** 2014-05-18

**Authors:** S. S. Jick, L. Li, G. J. Falcone, Z. P. Vassilev, M.-A. Wallander

**Affiliations:** 1 Boston Collaborative Drug Surveillance Program, Boston University School of Public Health, 11 Muzzey Street, Lexington, MA 02421 USA; 2 Department of Neurology, Massachusetts General Hospital, Boston, MA USA; 3 Department of Epidemiology, Harvard School of Public Health, Boston, MA USA; 4 Bayer HealthCare Pharmaceuticals, Whippany, NJ USA; 5 Department of Public Health and Caring Science, Uppsala University, Uppsala, Sweden

**Keywords:** Multiple sclerosis, Epidemiology, Cause of death, Cohort analysis, Mortality

## Abstract

We aimed to estimate rates, causes and risk factors of all-cause mortality in a large population-based cohort of multiple sclerosis (MS) patients compared with patients without MS. Using data from the UK General Practice Research Database, we identified MS cases diagnosed during 2001–2006 and validated using patients’ original records where possible. We also included MS cases during 1993–2000 identified and validated in an earlier study. Cases were matched to up to ten referents without MS by age, sex, index date (date of first MS diagnosis for cases and equivalent reference date for controls), general practice and length of medical history before first MS diagnosis. Patients were followed up to identify deaths; hazard ratios (HRs) and 95 % confidence intervals (CIs) were estimated using Cox-proportional regression. MS patients (*N* = 1,822) had a significantly increased risk of all-cause mortality compared with referents (*N* = 18,211); adjusted HR 1.7 (95 % CI 1.4–2.1). Compared with referents, female MS patients had a higher but not significantly different HR for death than males; adjusted HR 1.86 (95 % CI 1.46–2.38) vs. HR 1.31 (95 % CI 0.93–1.84), respectively. The most commonly recorded cause of death in MS patients was ‘MS’ (41 %), with a higher proportion recorded among younger patients. A significantly higher proportion of referents than MS patients had cancer recorded as cause of death (40 vs. 19 %). Patients with MS have a significant 1.7-fold increased risk of all-cause mortality compared with the general population. MS is the most commonly recorded cause of death among MS patients.

**Electronic supplementary material:**

The online version of this article (doi:10.1007/s00415-014-7370-3) contains supplementary material, which is available to authorized users.

## Introduction

Multiple sclerosis (MS) is a chronic, progressive neurological disorder, and is the major cause of non-traumatic disability in young adults [1]. Mortality rates are significantly higher in people with MS compared with the general population [2–4], yet causes of death and factors influencing survival in MS patients are not well understood, and further data addressing these questions are needed.

One important question is to what extent can the excess in mortality rates observed in MS patients be attributed to the disease. Another important question is what are the risk factors for the disease and what influence do they have on mortality. Identifying risk factors, and understanding their relative weight on the risk of mortality, is vital for improving our abilities to predict survival and for implementing a balanced therapeutic strategy. We therefore aimed to estimate mortality rates, describe cause-specific mortality and identify risk factors for mortality in a large population-based cohort of MS patients compared with patients without MS in the United Kingdom (UK).

## Methods

### Data source

We conducted a cohort study using prospectively collected data from the UK General Practice Research Database (GPRD). The GPRD comprises the anonymized computerized medical records of around 6 % of the UK population at any one time [5, 6]. The database is the core of the primary care data managed by the Clinical Practice Research Datalink (CPRD), and contains information on patients’ demographics, clinical events, and details of specialist referrals, hospital admissions and prescriptions. Additional information can be added as free text. Prescriptions are issued directly from the GPs computer, hence all are recorded. Patients in the GPRD are representative of the UK population with regard to age, sex and geographical distribution. Validation studies have demonstrated the quality of the data in the GPRD to be generally high [5], and the database has been used for several studies on MS [7–15]. The study protocol was approved by the CPRD Independent Scientific Advisory Committee for UK Medicines and Healthcare products Regulatory Agency database research.

### Study design and MS case ascertainment

We identified a cohort of incident and prevalent MS patients in the GPRD between January 1993 and December 2006. This was a two-step process involving two study periods. Firstly, we identified all possible and probable incident MS cases, and all prevalent MS cases occurring between January 2001 and December 2006. Secondly, we added to this 2001–2006 case set, all incident and prevalent MS cases identified and validated in our earlier studies conducted on the GPRD (between 1993 and 2000) [7, 8] from practices that were still contributing to the database in 2012. To identify incident MS cases between 2001 and 2006, we identified patients registered in the GPRD in this study period who had at least two continuous years of registration. Patients with a first recorded diagnosis for MS were identified as potential incident cases, and their computerized medical records were reviewed.

Potential incident cases were classified as either probable, possible, or unlikely MS using an overall clinical impression obtained after review by a neurologist (GJF) of all available data on diagnoses, treatments and referrals recorded in the patient record: *probable case*, multiple entries of MS diagnosis plus treatment and/or symptom codes; *possible case*, at least one entry of MS diagnosis plus codes for treatments or symptoms that may have been related to MS or multiple MS diagnoses with no supporting treatment or symptoms codes; *unlikely case*, one entry of MS diagnosis only and no supporting treatment or symptoms codes, or the record contained an alternate diagnosis such as amyotrophic lateral sclerosis.

Where available, patients’ original clinical paper records containing clinical notes, consultation letters, specialist referrals, test results and hospital discharge letters were retrieved, and were reviewed by a neurologist (GJF) to validate the MS diagnosis and classify the case. Each case was classified according to the McDonald criteria for MS [16] and categorized as definite, possible or unlikely. For cases where GP comments strongly indicated that the patient had MS but objective data were unavailable to fulfill the McDonald criteria, a fourth category called probable was used. Where there was uncertainty, a second neurologist reviewed the patient records and a final decision was reached by consensus. The index date was the date of MS diagnosis.

### Comparison group selection

Each case was randomly matched to up to 10 referent subjects without a recorded diagnosis of MS at any time in the GPRD by year of birth, sex, index date, general practice and length of medical history recorded before the index date. The index date for referents was the date of diagnosis of their matched MS case.

### Follow-up

Patients were followed up from the index date to identify deaths. The end of follow-up was the date of patient’s death, the date of transfer out of the practice, or the date of last data collection in the database (31 July 2012), whichever came first. The cause of death was ascertained from electronic and/or original clinical paper records. To supplement information on cause of death recorded in the GP records, we were able to link to death registry data for patients in practices in England, and we requested data from additional electronic GP notes where the cause of death was unknown, but there was an indication that additional electronic GP notes were available.

### Covariate information

Information on the following variables was extracted from the database: age at first MS diagnosis, sex, and lifestyle factors closest to and before the first MS diagnosis including smoking, body mass index (BMI) and alcohol abuse. Information on chronic comorbidities (recorded at any time in the database), acute illnesses (recorded at or within 1 year before diagnosis or any time after), and comedications (at or within 6 months before diagnosis or any time after) were also extracted. Comorbidities evaluated included both chronic diseases (chronic obstructive pulmonary disease or asthma, depression, diabetes, hypertension, heart disease and cancer) and acute illnesses [infections (respiratory, pneumonia/influenza, urinary tract, skin, eye or ear, or other), pain (joint, spine, muscle, migraine, eye or ear) and dyspepsia]. Comedications evaluated included antibiotics, antidepressants, skeletal muscle relaxants, antipsychotics, anti-Parkinson’s drugs, anticonvulsants, opioids, non-steroidal anti-inflammatory drugs (NSAIDs), topical NSAIDs, other analgesics, proton pump inhibitors, steroids, topical steroids, statins, and oral contraceptives. We also calculated the Charlson Comorbidity Index [17] before the first recorded MS diagnosis. We were unable to obtain information on use of interferon beta because, in the UK, it is mostly prescribed in secondary care and is not always captured in GP records.

For the 902 MS patients with original clinical records, we retrieved additional information on MS subtype [relapsing-remitting MS (RRMS), primary progressive MS (PPMS), secondary progressive MS (SPMS) or unknown], and MS symptoms at onset. These were classified into four main groups: sensory, motor, optic neuritis and ‘other’. The latter group included other optic and visual anomalies, other cranial nerve anomalies, dysarthria and asthenia. Patients without symptoms recorded were treated as a separate group with missing symptoms.

### Statistical analyses

All definite, probable and possible MS cases were included in the analyses. Patient characteristics were described at or before the index date and separately at the end of follow-up using counts [percentages (%)] for discrete and categorical variables, and means [standard deviation (SD)] for continuous variables. We compared patients’ characteristics including lifestyle risk factors, comorbidities and comedications between the MS patients and referents. For continuous variables, we used *t* tests; for categorical variables, we used Chi square tests or Fisher’s exact tests where necessary. Crude death rates with 95 % confidence intervals (CIs) were calculated overall and stratified by age at first diagnosis, sex and type of MS. Cause of death was described where possible. Survival probabilities for fixed categorical variables related to all-cause mortality were estimated using Kaplan–Meier survival analyses, both overall and stratified by age at first diagnosis and sex. Hazard ratios (HRs) and 95 % CIs for all-cause mortality were estimated using Cox-proportional regression models adjusted for potential confounding variables. Statistical analyses were performed using SAS version 9.2 (SAS Institute, Cary, NC).

## Results

Between January 2001 and December 2006, 1278 incident and 63 prevalent MS cases were identified. In addition, 435 incident and 46 prevalent MS cases identified between 1993 and 2000 were retrieved from previous studies [7–10, 12–14], giving a total of 1,822 MS cases (1,507 definite or probable and 315 possible), matched to 18,211 referents. Nearly, three quarters of MS cases were female and the mean age at diagnosis was 42.1 years. PPMS patients were generally older at diagnosis than RRMS patients (mean age 50 vs. 40 years; *p* < 0.001), and more likely to be male (42.4 vs. 25.1 %; *p* < 0.001) and current smokers (32 vs. 29.4 %, *p* = 0.30) (Table 1). The length of follow-up post-cohort entry date was similar for MS patients and their non-MS comparators: 7.9 years (range 1 day–19 years).

**Table 1:** Basic characteristics of MS cases and matched referent subjects at cohort entry, overall and stratified by type of MS

Characteristic	MS cases *N* = 1,822 [*n* (%)]	Referents *N* = 18,211 [*n* (%)]	RRMS^a^ *N* = 769 [*n* (%)]	Referents *N* = 7,690 [*n* (%)]	PPMS *N* = 125 [*n* (%)]	Referents *N* = 1,250 [*n* (%)]
Mean age (years) at cohort entry (index date; SD)	42.08 (11.79)	42.0 (11.72)	40.01 (11.14)	39.94 (11.15)	49.98 (10.43)	49.92 (10.39)
Sex						
Males	481 (26.40)	4,801 (26.36)	193 (25.10)	1,930 (25.10)	53 (42.40)	530 (42.40)
Females	1,341 (73.60)	13,410 (73.64)	576 (74.90)	5,760 (74.90)	72 (57.60)	720 (57.60)
Smoking status						
Current*^,†^	569 (31.23)	4,363 (23.96)	226 (29.39)	1,902 (24.73)	40 (32.00)	289 (23.12)
Former	239 (13.12)	2,390 (13.12)	101 (13.13)	963 (12.52)	23 (18.40)	199 (15.92)
Never	777 (42.65)	8,921 (48.99)	349 (45.38)	3,807 (49.51)	50 (40.00)	595 (47.60)
Unknown	237 (13.01)	2,537 (13.93)	93 (12.09)	1,018 (13.24)	12 (9.60)	167 (13.36)
BMI (kg/m^2^)						
<18.5	46 (2.52)	379 (2.08)	17 (2.21)	179 (2.33)	2 (1.60)	18 (1.44)
18.5–24.99	706 (38.75)	6,948 (38.15)	301 (39.14)	2,980 (38.75)	46 (36.80)	408 (32.64)
25.0–29.99	426 (23.38)	4,100 (22.51)	187 (24.32)	1,676 (21.79)	39 (31.20)	339 (27.12)
≥30	248 (13.61)	2,406 (13.21)	112 (14.56)	993 (12.91)	12 (9.60)	198 (15.84)
Unknown	396 (21.73)	4,378 (24.04)	152 (19.77)	1,862 (24.21)	26 (20.80)	287 (22.96)
Alcohol abuse^†^	25 (1.37)	370 (2.03)	3 (0.39)	133 (1.73)	5 (4.0)	50 (4.0)
Mean length of recorded medical history (years; SD)						
Before index date	7.86 (4.44)	7.97 (4.43)	7.93 (4.50)	8.03 (4.49)	8.83 (4.51)	9.02 (4.52)
Follow-up after index date^†^	7.85 (4.50)	7.95 (4.49)	8.95 (4.33)	8.18 (4.40)	8.15 (4.32)	8.15 (4.08)

For the 894 MS patients whose type of MS was determined through original clinical records

*BMI* body mass index, *MS* multiple sclerosis, *PPMS* primary progressive MS, *RRMS* relapsing-remitting MS, *SD* standard deviation

* *p* < 0.05 for comparison between patients with MS and matched referent subjects (for all MS patients)

^†^ *p* < 0.05 for comparison between patients with RRMS and matched referent subjects

^a ^Includes 64 patients with secondary progressive MS

### Characteristics of MS vs. referent subjects

There were a number of significant differences between the characteristics, comorbidities and comedications of MS patients and their matched referents (Tables 1, 2, 3). At the index date, MS patients were more likely to be current smokers (*p* < 0.05). Within the year before the index date, MS patients overall were more likely to have had a urinary tract infection (*p* < 0.05), PPMS cases were more likely to have had an acute respiratory infection (*p* < 0.05) and RRMS cases were more likely to have had other infections (*p* < 0.05). Both RRMS and PPMS patients were more likely to have a history of depression (*p* < 0.05), and to have received, antidepressants, anticonvulsants, opioids, muscle relaxants and anti-Parkinson’s drugs at or within the 6 months before the index date (*p* < 0.05 for all). In addition, during this time period, MS patients overall were more likely to have received statins, NSAIDs, systemic glucocorticoids, antibiotics and antipsychotics (*p* < 0.05 for all). Compared with RRMS patients, PPMS patients were more likely to receive symptomatic MS treatment (e.g., muscle relaxants; 16.0 vs. 6.5 %; *p* = 0.0002), but were less likely to receive antibiotics (12.8 vs. 23.3 %; *p* = 0.0085).

**Table 2:** Comorbidities of MS cases and matched referent subjects at cohort entry, overall and stratified by type of MS

Characteristic	MS cases *N* = 1,822 [*n* (%)]	Referents *N* = 18,211 [*n* (%)]	RRMS^a^ cases *N* = 769 [*n* (%)]	Referents *N* = 7,690 [*n* (%)]	PPMS cases *N* = 125 [*n* (%)]	Referents *N* = 1,250 [*n* (%)]
Chronic comorbidities^b^						
COPD and asthma	302 (16.58)	2,890 (15.87)	117 (15.21)	1,245 (16.19)	18 (14.40)	197 (15.76)
Depression*^,†,§^	508 (27.88)	3,677 (20.19)	188 (24.45)	1,529 (19.88)	45 (36.00)	238 (19.04)
Diabetes	35 (1.92)	390 (2.14)	13 (1.69)	141 (1.83)	3 (2.40)	44 (3.52)
Hypertension	137 (7.52)	1,544 (8.48)	47 (6.11)	538 (7.00)	13 (10.40)	179 (14.32)
Heart disease	34 (1.87)	439 (2.41)	8 (1.04)	119 (1.55)	4 (3.20)	64 (5.12)
Cancer	50 (2.74)	558 (3.06)	15 (1.95)	223 (2.90)	5 (4.00)	50 (4.00)
Acute comorbidities^c^						
Acute respiratory infection^§^	252 (13.83)	2,673 (14.68)	113 (14.69)	1,143 (14.86)	9 (7.20)	171 (13.68)
Pneumonia and influenza	18 (0.99)	169 (0.93)	11 (1.43)	70 (0.91)	1 (0.80)	14 (1.12)
Urinary tract infection*	99 (5.43)	683 (3.75)	36 (4.68)	284 (3.69)	8 (6.40)	41 (3.28)
Skin infection	164 (9.00)	1,566 (8.60)	68 (8.84)	652 (8.48)	5 (4.00)	96 (7.68)
Eye or Ear infection	4 (0.22)	33 (0.18)	3 (0.39)	15 (0.20)	0 (–)	0 (–)
Other infection*^,†^	149 (8.18)	1,256 (6.90)	69 (8.97)	533 (6.93)	5 (4.00)	73 (5.84)
Dyspepsia	35 (1.92)	282 (1.55)	11 (1.43)	120 (1.56)	2 (1.60)	23 (1.84)
Charlson Comorbidity Index at cohort entry						
Low (0)	1,393 (76.45)	14,192 (77.93)	615 (79.97)	6,048 (78.65)	96 (76.80)	931 (74.48)
Medium (1–2)	405 (22.23)	3,822 (20.99)	147 (19.12)	1,574 (20.47)	26 (20.80)	296 (23.68)
High (>2)	24 (1.32)	197 (1.08)	7 (0.91)	68 (0.88)	3 (2.40)	23 (1.84)

For the 894 MS patients whose type of MS was determined through original clinical records

*COPD* chronic obstructive pulmonary disorder, *MS* multiple sclerosis, *PPMS* primary progressive MS, *RRMS* relapsing-remitting MS

* *p* < 0.05 for comparison between patients with MS and matched referent subjects (for all MS patients)

^†^ *p* < 0.05 for comparison between patients with RRMS and matched referent subjects

^§^ *p* < 0.05 for comparison between patients with PPMS and matched referent subjects

^a ^Includes 64 patients with secondary progressive MS

^b ^Ever before, or at cohort entry

^c ^During the year before, or at index date

**Table 3:** Comedications of MS cases and matched referent subjects at cohort entry, overall and stratified by type of MS

Characteristic	All MS cases *N* = 1,822 [*n* (%)]	Referents *N* = 18,211 [*n* (%)]	RRMS^a^ cases *N* = 769 [*n* (%)]	Referents *N* = 7,690 [*n* (%)]	PPMS cases *N* = 125 [*n* (%)]	Referents *N* = 1,250 [*n* (%)]
Comedications (at cohort entry [index date] or within the 6 months before)						
Systemic glucocorticoids*^,†^	180 (9.88)	713 (3.92)	69 (8.97)	288 (3.75)	8 (6.40)	52 (4.16)
Antidepressants*^,†,§^	380 (20.86)	1,582 (8.69)	132 (17.17)	662 (8.61)	28 (22.40)	102 (8.16)
Anticonvulsants*^,†,§^	122 (6.70)	225 (1.24)	40 (5.20)	88 (1.14)	11 (8.80)	25 (2.00)
Antidiabetics	24 (1.32)	283 (1.55)	12 (1.56)	112 (1.46)	0 (–)	30 (2.40)
Opioids*^,†,§^	345 (18.94)	1,585 (8.70)	122 (15.86)	621 (8.08)	23 (18.40)	133 (10.64)
NSAIDs*^,†^	316 (17.34)	1,806 (9.92)	122 (15.86)	710 (9.23)	21 (16.80)	152 (12.16)
Statins*	61 (3.35)	447 (2.45)	20 (2.60)	137 (1.78)	4 (3.20)	75 (6.00)
Antibiotics*^,†^	403 (22.12)	3,598 (19.76)	179 (23.28)	1,546 (20.10)	16 (12.80)	227 (18.16)
Muscle relaxants*^,†,§^	173 (9.50)	399 (2.19)	50 (6.50)	153 (1.99)	20 (16.00)	37 (2.96)
Antipsychotics*^,†^	122 (6.70)	387 (2.13)	54 (7.02)	160 (2.08)	5 (4.00)	20 (1.60)
Anti-Parkinson drugs*^,†,§^	23 (1.26)	54 (0.30)	8 (1.04)	17 (0.22)	3 (2.40)	4 (0.32)
PPIs*	83 (4.56)	657 (3.61)	24 (3.12)	243 (3.16)	6 (4.80)	68 (5.44)

For the 894 MS patients whose type of MS was determined through original clinical records

*MS* multiple sclerosis, *NSAIDs* non-steroidal anti-inflammatory drugs, *PPMS* primary progressive MS, *PPIs* proton pump inhibitors, *RRMS* relapsing-remitting MS

* *p* < 0.05 for comparison between patients with MS and matched referent subjects (for all MS patients)

^†^ *p* < 0.05 for comparison between patients with RRMS and matched referent subjects

^§^ *p* < 0.05 for comparison between patients with PPMS and matched referent subjects

^a ^Includes 64 patients with secondary progressive MS

Compared with referents, at the end of follow-up MS patients were more likely to be smokers (26.3 vs. 22.0 %) to have a BMI <18.5 kg/m^2^ (3.6 vs. 2.0 %), a history of depression (45.7 vs. 29.9 %), more infections, including pneumonia and influenza (1.8 vs. 0.9 %), urinary tract infections (10.5 vs. 3.9 %), and skin infections (11.9 vs. 9.8 %), and to have received more comedications in the prior 6 months (12.3 vs. 8.5 %) (*p* < 0.05 for all) (Online Resource 1).

### Mortality rates

Of the 1,822 MS cases, 130 (7.1 %) died during 14,295 person-years of follow-up, while 573 (3.1 %) referents died during 144,760 person-years of follow-up. The crude death rate for MS patients was 9.1 (95 % CI 7.6–10.8) per 1,000 person-years compared with 4.0 (95 % CI 3.6–4.3) per 1,000 person-years for the non-MS referents. Mortality rates were higher in MS patients compared with their matched referents in each age group and for both males and females. Patients with PPMS had an almost twofold greater mortality rate compared with RRMS patients (11.8 vs. 5.8 per 1,000 person-years). However, these death rates were similarly higher compared with their corresponding referents; 6.2 vs. 2.9 per 1,000 person-years for PPMS and RRMS referents, respectively) (Table 4).

**Table 4:** Death rate per 1,000 person-years in patients with MS and matched referent subjects overall, stratified by age at first diagnosis of MS, sex, and type of MS

	Deaths (*N*)	Population size	Person-years	Death rates (95 % CI)
Overall				
Patients with MS	130	1,822	14,295	9.09 (7.63–10.76)
Referent subjects	573	18,211	144,760	3.96 (3.64–4.29)
Age group at diagnosis (years)				
<30				
Patients with MS	8	264	1,996	4.01 (1.86–7.61)
Referent subjects	5	2,666	18,857	0.27 (0.10–0.59)
30–39				
Patients with MS	20	522	4,036	4.96 (3.11–7.52)
Referent subjects	48	5,217	40,721	1.18 (0.88–1.55)
40–49				
Patients with MS	35	564	4,600	7.61 (5.38–10.46)
Referent subjects	116	5,634	47,483	2.44 (2.03–2.92)
50–59				
Patients with MS	30	338	2,744	10.93 (7.51–15.41)
Referent subjects	180	3,380	27,643	6.51 (5.61–7.52)
≥60				
Patients with MS	37	134	919	40.26 (28.76–54.91)
Referent subjects	224	1,314	10,057	22.27 (19.50–25.34)
Sex				
Male				
Patients with MS	41	481	3,717	11.03 (8.02–14.82)
Referent subjects	228	4,801	37,284	6.12 (5.36–6.95)
Female				
Patients with MS	89	1,341	10,578	8.41 (6.80–10.30)
Referent subjects	345	13,410	107,476	3.21 (2.88–3.56)
Type of MS				
RRMS				
Patients with MS	40	769^a,b^	6,881	5.81 (4.21–7.84)
Referent subjects	183	7,690	62,928	2.91 (2.51–3.35)
PPMS				
Patients with MS	12	125^a^	1,018	11.79 (6.39–20.04)
Referent subjects	63	1,250	10,184	6.19 (4.79–7.86)

*CI* confidence interval, *MS* multiple sclerosis, *RRMS* relapsing-remitting MS, *PPMS* primary progressive MS

^a ^For the 894 MS cases confirmed by original clinical records

^b ^Including 64 patients with SPMS

**Fig. 1:**
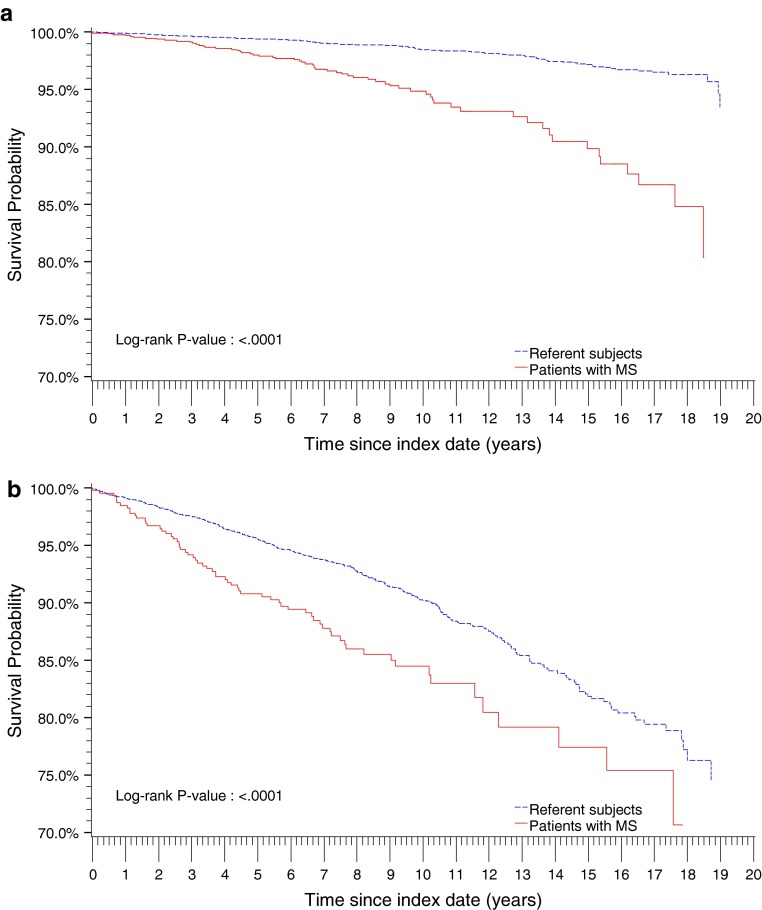
**a** Kaplan–Meier plot for the survival probabilities (all-cause mortality) of patients <50 years at index date. *MS* multiple sclerosis. **b** Kaplan–Meier plot for the survival probabilities (all-cause mortality) of patients ≥50 years at index date. *MS* multiple sclerosis

Patients aged ≥50 years at diagnosis had shorter survival than those aged <50 years at diagnosis (Fig. 1). For the latter group, an increasing difference in survival between MS cases and their matched non-MS referents was observed with increasing length of follow-up. Survival probabilities were similar for both male and female MS patients, with 10-year survival at 90 and 93 %, and 15-year survival at 86 and 87 % for males and females, respectively.

### Cause of death

Among MS patients who died during follow-up, the most commonly recorded cause of death was MS (40.8 %), followed by pneumonia (25.4 %), cancer (18.5 %), cardiovascular disease (13.9 %) and non-infectious respiratory diseases (10.0 %). Among deceased referents, cause of death was recorded as pneumonia in 6.8 %, cancer in 39.8 % and cardiovascular disease in 19.4 %. The higher proportion of cancer deaths among referents compared with the MS patients (*p* < 0.0001) is noteworthy. No material difference was found in the proportion of deaths recorded as suicide or accident between MS patients and referents; 1.5 and 1.9 %, respectively. However, these proportions are based on only two MS patients (one suicide) and 11 referents (two suicides). Additional data on cause of death are provided in Online Resources 2 and 3.

Among deceased MS patients, more males than females had their cause of death recorded as cardiovascular disease (24.4 vs. 9.0 %, *p* = 0.02) or MS (43.9 vs. 39.3 %, *p* = 0.62), while more females than males had cancer recorded as cause of death (20.2 vs. 14.6 %, *p* = 0.45). Patients older at diagnosis (≥50 years) had a higher proportion of deaths recorded as due to cancer compared with patients diagnosed at a younger age (<50 years) (22.4 vs. 14.3 %, *p* = 0.23), while patients younger at diagnosis had a higher proportion of deaths recorded as due to MS (47.6 vs. 34.3 %, *p* = 0.12) or pneumonia (31.8 vs. 19.4 %, *p* = 0.11).

### Risk factors for mortality

Compared with referents, MS patients had a significantly increased risk of all-cause mortality; adjusted HR 1.68 (95 % CI 1.38–2.05) (Table 5). Age was a strong effect modifier, with the youngest MS patients yielding the highest adjusted HR 13.2 (95 % CI 4.2–41.7) for patients aged <30 years at diagnosis. Compared with referents, female MS patients had a higher overall HR for death than male MS patients, although the HRs were not significantly different; adjusted HR 1.86 (95 % CI 1.46–2.38) vs. HR 1.31 (95 % CI 0.93–1.84). While we observed a significantly higher HR for RRMS patients compared to referents [adjusted HR 1.50 (95 % CI 1.06–2.14)], a significantly higher HR was not found for PPMS patients [adjusted HR 1.32 (95 % CI 0.69–2.55)]. It should be noted, however, that the number of PPMS patients was small.

**Table 5:** Hazard ratios and 95 % confidence intervals for all-cause mortality among MS cases versus matched referent subjects

Characteristic	Death in MS patients *N* = 130 [*n* (%)]	Deaths in non-MS referents *N* = 573 [*n* (%)]	HR (95 % CI)	
			Model 1^a^	Model 2^b^
Overall	130 (100)	573 (100)	2.30 (1.90–2.78)	1.68 (1.38–2.05)
Age at first MS diagnosis (years)				
<30	8 (6.2)	5 (0.87)	14.19 (4.62–43.54)	13.18 (4.17–41.67)
30–39	20 (15.4)	48 (8.4)	4.27 (2.53–7.19)	3.29 (1.93–5.61)
40–49	35 (26.9)	116 (20.2)	3.13 (2.15–4.57)	2.16 (1.45–3.21)
50–59	30 (23.1)	180 (31.4)	1.66 (1.13–2.44)	1.33 (0.88–1.99)
≥60	37 (28.5)	224 (39.1)	1.82 (1.28–2.57)	1.47 (1.03–2.12)
Sex				
Male	41 (31.5)	228 (39.8)	1.79 (1.28–2.49)	1.31 (0.93–1.84)
Female	89 (68.5)	345 (60.2)	2.63 (2.09–3.32)	1.86 (1.46–2.38)
Type of MS^c^				
RRMS^d^	40 (30.8)	183^e^ (31.9)	1.94 (1.38–2.73)	1.50 (1.06–2.14)
PPMS	12 (9.2)	63^f^ (11.0)	1.89 (1.02–3.51)	1.32 (0.69–2.55)

*CI* confidence interval, *HR* hazard ratio, *MS* multiple sclerosis, *PPMS* primary progressive MS, *RRMS* relapsing-remitting MS, *SPMS* secondary progressive MS

^a ^Adjusted for matching variables (age, sex, index date, GP, and length of medical history recorded before the index date)

^b ^Adjusted for smoking and comedications (including antidepressants, anticonvulsants, opioids, muscle relaxants) within 6 months before or at the index date, in addition to the matching variables

^c ^MS cases confirmed by original clinical paper records

^d ^Including patients with SPMS

^e ^Matched to patients with RRMS

^f ^Matched to patients with PPMS

## Discussion

This large study of 1,822 people with MS in UK primary care provides national estimates of all-cause mortality among this patient group. We observed a 1.7-fold increased risk of all-cause mortality for MS patients compared with the general population. This increase in risk is in line with reports from other large population-based cohorts [2–4, 18–21], although direct comparisons are difficult owing to differences in geographical regions and/or study periods.

Consistent with other reports, MS was the main recorded cause of death (40.8 %) in MS patients in our study. This proportion is lower than that in other studies, albeit from a wide range of geographical populations, where MS was the reported cause of death in more than 50 % of affected patients [4]. Respiratory diseases, infections, cardiovascular diseases and cancers are other commonly reported causes of death in patients with MS [2, 22], and our results are consistent with these findings. It should be noted that the “real” causes of death in patients with MS are not always recorded in patient records or registries. While it is accepted that MS is not per se a fatal disease, neither our study nor prior studies have been able to identify all direct causes of death in these patients. Thus, complete information on cause of death remains unattainable. The lower proportion of MS patients with cancer as the cause of death compared to non-MS referents could be due to increased susceptibility to acute causes of death (such as infections or acute cardiovascular events). These acute and sometimes fatal comorbidities may act as competing risks, truncating the long latency periods needed for a neoplastic disease to express clinically. Current evidence suggests that frequency of suicides may be higher in MS patients [23]. In only three patients was suicide the recorded cause of death in our study (one MS patient and two referents), which was insufficient to detect any material difference between the patient groups.

The progressive nature of MS is reflected in our study in the increasing differences in all-cause mortality rates seen between MS patients and their matched non-MS referents with increasing length of follow-up. It is interesting that all HRs were attenuated when risk factors for comedications associated with MS were included in the model. This suggests that some of the increased risk for death is associated with the comorbidities and resulting comedications that occur more frequently in patients with MS. It is notable that the HRs for death in the MS cohort compared to the non-MS cohort were highest in the younger patients and decreased with increasing age. It is possible that the decreased baseline risk of death in younger people contributed to the higher HR, while baseline risks increased for all patients with increasing age leading to lower HRs. We did not, however, find any statistically significant difference in survival between males and females, although the adjusted HR for all-cause mortality was higher for females. Other reports concerning gender difference in survival have been mixed [2, 4, 18, 19].

MS patients in this study were more likely to have been diagnosed with depression in the period between first MS symptoms and first MS diagnosis. Diagnoses of depression in the years before first symptoms were similar in the MS patients and their matched non-MS referents. Given their timing, these findings can be interpreted either as “reactive” in the setting of a neurological disease of uncertain etiology, or as a direct result of neural damage caused by the ongoing inflammatory process affecting the CNS. In any case, these results highlight that depression, together with the heavy burden it imposes on functional status, becomes a relevant problem in MS subjects long before a final diagnoses of MS can be reached. The higher incidence of urinary tract infection in the months prior to MS diagnosis is consistent with decreased mobility found in MS patients, particularly those with PPMS. While the incidence of Parkinson’s disease was higher in the MS group compared to the non-MS referents, the absolute number of cases was small. Previous reports have not found similar differences. It is possible that at least some of the 23 cases had Parkinsonian symptoms due to compromise of the basal ganglia by the underlying neuro-inflammatory process, but were misdiagnosed and treated as idiopathic Parkinson’s disease. Alternatively, the association could be explained by unmeasured confounders.

Strengths of our study include the large sample size, the high quality of the database and the controlled study design. The representativeness of the study population means that the results can be generalized to the UK as whole, thus the study has high external validity. In addition, for some MS cases, we were able to validate the diagnosis by accessing patients’ original clinical records, which along with linkage to death registry data, enabled additional clinical information to be obtained. We were also able to obtain and analyze data on a large number of potential risk factors for mortality.

Our study also has limitations. We were unable to validate the MS diagnosis via original clinical records for all patients, thus it is likely that some misclassification of MS cases occurred. Any misclassification would likely have been random, non-differential, and would have had little effect on the mortality rates in the study. Any effects on the HRs would likely have been small and biased toward the null. However, when we repeated the main analyses for cases whose diagnosis was made on original clinical records and for cases whose diagnosis was made from electronic records only, we found the results from the two groups to be consistent. When we repeated the analyses again, restricted to definite and probable MS patients, the results were similar to the full group analysis (Online Resources 4–7). We were also unable to identify cause of death for around 15 % of deceased MS patients and 17 % of deceased referents, which may have influenced comparisons of cause of death. However, we found the most common causes of deaths were consistent with those reported elsewhere [22]. Our study was underpowered to explore risk factors for cause-specific mortality, and it is also possible that there was some underestimation of our reported all-cause mortality rates. This is because follow-up time may not have been long enough to observe mortality, especially for MS cases identified between 2001 and 2006. It is also possible that a small number of deaths were not recorded by the GP though recording of death is required of all GPs and this number should be minimal. In addition, we were unable to evaluate the influence of interferon beta, which has been shown to slow disease progression [24–26] and reduce all-cause mortality [26–28] in patients with RRMS. Although this may have affected our estimates of all-cause mortality among RRMS patients, it is unlikely to have affected the HRs associated with factors found to be predictive of all-cause mortality, as such confounding by indication is unlikely.

In summary, our findings show that patients with MS have increased all-cause mortality rates compared with the general population, and that some of this increase is likely related to the comorbidities that occur more frequently in patients with MS. Most deaths in MS patients are recorded as due to the disease itself.

## Electronic supplementary material

Below is the link to the electronic supplementary material.

Supplementary material 1 (DOC 60 kb)

Supplementary material 2 (DOC 83 kb)

Supplementary material 3 (DOC 100 kb)

Supplementary material 4 (DOC 33 kb)

Supplementary material 5 (DOC 31 kb)

Supplementary material 6 (DOC 30 kb)

Supplementary material 7 (DOC 38 kb)

Supplementary material 8 (DOC 34 kb)
