# Supplementary material for: Mortality of patients with multiple sclerosis: a cohort study in UK primary care
Source: J Neurol. 2014 May 18;261(8):1508–17. doi: 10.1007/s00415-014-7370-3 (PMC4119255; doi:10.1007/s00415-014-7370-3)
Supplement: Supplementary file 1 — Supplementary material 1 (DOC 60 kb) [file 415_2014_7370_MOESM1_ESM.doc]

**Mortality of Patients with Multiple Sclerosis:
A Cohort Study in UK Primary Care**

SS Jick, L Li, GJ Falcone, ZP Vassilev, M-A Wallander

Corresponding author: Susan Jick DSc, Boston Collaborative Drug Surveillance Program, Boston University School of Public Health, 11 Muzzey Street, Lexington, MA 02421

Telephone: 781-862-6660; Fax: 781-862-1680; email: [sjick@bu.edu](mailto:sjick@bu.edu)

Characteristics of patients with MS and matched referent subjects at end of follow-up

|  | **Patients with MS**  **N=1822**  **(n [%])** | **Referent subjects**  **N = 18211**  **(n [%])** |
| --- | --- | --- |
| **Age at end of follow-up (years)**  Mean (SD) | 49.96 (12.59) | 50.00 (12.79) |
| < 30 | 83 (4.56) | 939 (5.16) |
| 30 – 39 | 311 (17.07) | 3038 (16.68) |
| 40 – 49 | 500 (27.44) | 4904 (26.93) |
| 50 – 59 | 493 (27.06) | 4963 (27.25) |
| ≥ 60 | 435 (23.87) | 4367 (23.98) |
| **Smoking^a^**  Current | 479 (26.29) | 4001 (21.97) |
| Former | 521 (28.59) | 4727 (25.96) |
| Never | 789 (43.30) | 8905 (48.90) |
| Unknown | 33 (1.81) | 578 (3.17) |
| **Body mass index^a^ (kg/m^2^)**  < 18.5 | 65 (3.57) | 358 (1.97) |
| 18.5 – 24.99 | 688 (37.76) | 6529 (35.85) |
| 25.0 – 29.99 | 504 (27.66) | 5188 (28.49) |
| ≥ 30 | 364 (19.98) | 4019 (22.07) |
| Unknown | 201 (11.03) | 2117 (11.62) |
| **Alcohol abuse^a^** | 44 (2.41) | 599 (3.29) |
| **Length of medical history recorded before end of follow-up ( year; mean [SD])** | 15.70 (5.79) | 15.92 (5.76) |
| **Charlson Comorbidity Index at end of  follow-up**  Low (0) | 1194 (65.53) | 12299 (67.54) |
| Medium (1-2) | 520 (28.54) | 4920 (27.02) |
| High (>2) | 108 (5.93) | 992 (5.45) |
| **Chronic comorbidities, ever before or  at end of follow-up**  COPD & asthma | 361 (19.81) | 3612 (19.83) |
| Depression^a^ | 833 (45.72) | 5450 (29.93) |
| Diabetes | 102 (5.60) | 946 (5.19) |
| Hypertension^a^ | 277 (15.20) | 3234 (17.76) |
| Heart disease | 70 (3.84) | 784 (4.31) |
| Cancer | 124 (6.81) | 1322 (7.26) |
| **Acute comorbidities, one year before or at end of follow-up**  Acute respiratory infection | 237 (13.01) | 2439 (13.39) |
| Pneumonia & Influenza^a^ | 32 (1.76) | 167 (0.92) |
| Urinary tract infection^a^ | 192 (10.54) | 702 (3.85) |
| Skin infection^a^ | 216 (11.86) | 1785 (9.80) |
| Eye or Ear infection | 2 (0.11) | 20 (0.11) |
| Other infection | 154 (8.45) | 1161 (6.38) |
| Dyspepsia | 29 (1.59) | 346 (1.90) |
| **Co-medications, 6 months before or at end of follow-up**  Systemic glucocorticoids^a^ | 224 (12.29) | 1538 (8.45) |
| Antidepressants^a^ | 693 (38.04) | 2768 (15.20) |
| Anticonvulsants^a^ | 423 (23.22) | 557 (3.06) |
| Antidiabetics | 73 (4.01) | 714 (3.92) |
| Opioids^a^ | 474 (26.02) | 2665 (14.63) |
| NSAIDs^a^ | 273 (14.98) | 2264 (12.43) |
| Statins | 198 (10.87) | 2017 (11.08) |
| Antibiotics^a^ | 624 (34.25) | 4422 (24.28) |
| Muscle relaxants ^a^ | 492 (27.00) | 735 (4.04) |
| Antipsychotics^a^ | 87 (4.77) | 597 (3.28) |
| Anti-Parkinson drugs^a^ | 95 (5.21) | 87 (0.48) |
| PPIs^a^ | 338 (18.55) | 2518 (13.83) |

^a^P < 0.05 for comparison between patients with MS and matched referent subjects
