# Supplementary material for: Mortality of patients with multiple sclerosis: a cohort study in UK primary care
Source: J Neurol. 2014 May 18;261(8):1508–17. doi: 10.1007/s00415-014-7370-3 (PMC4119255; doi:10.1007/s00415-014-7370-3)
Supplement: Supplementary file 2 — Supplementary material 2 (DOC 83 kb) [file 415_2014_7370_MOESM2_ESM.doc]

**Mortality of Patients with Multiple Sclerosis:
A Cohort Study in UK Primary Care**

SS Jick, L Li, GJ Falcone, ZP Vassilev, M-A Wallander

Corresponding author: Susan Jick DSc, Boston Collaborative Drug Surveillance Program, Boston University School of Public Health, 11 Muzzey Street, Lexington, MA 02421

Telephone: 781-862-6660; Fax: 781-862-1680; email: [sjick@bu.edu](mailto:sjick@bu.edu)

**Causes of death among patients diagnosed with MS during the years 1993 to 2000 who subsequently died**

| Patient  No. | MS Diagnosis Date | Year of Birth | Sex | Date of Death | Cause of death |
| --- | --- | --- | --- | --- | --- |
| 1 | 1/5/1996 | 1966 | F | 3/26/2006 | MS, pneumonia |
| 2 | 5/25/1995 | 1952 | F | 10/2/2002 | Colon Cancer |
| 3 | 7/15/1997 | 1945 | M | 2/6/1999 | septicemia, liver disease |
| 4 | 4/6/1993 | 1958 | M | 8/25/1999 | Pneumonia |
| 5 | 1/13/1993 | 1949 | F | 10/19/2006 | MS, Pneumonia |
| 6^a^ | 8/2/1993 | 1926 | M | 10/1/2003 | No cause noted |
| 7 | 8/25/1993 | 1948 | M | 4/18/2000 | IHD, LVF, MS |
| 8 | 11/28/1994 | 1935 | F | 1/2/2009 | Breast cancer |
| 9 | 9/21/1995 | 1914 | M | 10/9/1997 | Kidney cancer, stroke |
| 10 | 5/22/1997 | 1945 | F | 12/27/2004 | Ovarian cancer |
| 11 | 9/29/1997 | 1925 | M | 1/6/2001 | IHD |
| 12 | 9/18/1995 | 1927 | F | 5/18/1999 | Pneumonia, MS |
|  |  |  |  |  |  |
| 13 | 1/10/1998 | 1954 | M | 7/25/2005 | Stroke |
| 14 | 8/11/1995 | 1958 | F | 4/22/2008 | MS, pneumonia |
| 15 | 10/6/1994 | 1939 | M | 3/27/1999 | UTI, septicemia |
| 16 | 2/12/1996 | 1950 | M | 7/29/2000 | Insect bite, drowning, MS |
| 17 | 12/13/1993 | 1947 | F | 8/29/1998 | CVD, pneumonia, MS |
| 18 | 6/1/1994 | 1948 | F | 4/28/2008 | MS, respiratory failure |
| 19 | 11/20/1995 | 1940 | F | 2/21/1997 | Lung cancer |
| 20 | 1/1/1994 | 1946 | M | 2/26/2007 | MI, MS, IHD |
| 21 | 1/25/1995 | 1946 | F | 9/5/2008 | Intestinal obstruction |
| 22^a^ | 7/27/1994 | 1955 | F | 8/28/2005 | Lung cancer |
| 23 | 8/3/1995 | 1945 | F | 2/26/2007 | Lung cancer |
| 24 | 12/15/1995 | 1947 | F | 2/25/2002 | PE post surgery, MS, OA hip |
| 25 | 5/25/1993 | 1934 | F | 1/18/1998 | Choking |
| 26 | 1/13/1997 | 1949 | F | 3/17/1998 | Vascular insufficiency intestine, portal vein thrombosis |
| 27 | 1/10/1995 | 1954 | M | 10/30/2005 | Lung cancer |
| 28 | 5/7/1993 | 1932 | F | 12/26/1993 | Pneumonia |
| 29 | 11/17/1997 | 1964 | M | 6/15/2006 | MS |
| 30 | 6/1/1995 | 1957 | F | 9/29/2010 | MS |
| 31^a^ | 8/26/1995 | 1928 | F | 1/16/2000 | Cause unknown |
| 32 | 10/13/1994 | 1963 | M | 2/21/2010 | Cause unknown |
| 33 | 1/31/1995 | 1973 | F | 9/13/2004 | Pneumonia |
| 34 | 11/25/1993 | 1941 | F | 12/20/2011 | MS, pneumonia |
| 35 | 5/18/1998 | 1949 | M | 2/5/2002 | Pneumonia, neurofibromatosis |
| 36 | 9/11/2000 | 1935 | F | 5/7/2003 | Cancer |
| 37^a^ | 3/23/1993 | 1958 | F | 11/5/2010 | MS, convulsions |
| 38 | 10/13/1994 | 1932 | F | 12/31/1994 | Cause unknown |
| 39 | 2/15/2000 | 1972 | F | 6/12/2010 | MS, choking |
| 40 | 4/20/1998 | 1953 | F | 3/7/2006 | Cancer |
| 41 | 7/3/1998 | 1965 | F | 2/8/2000 | Suicide |
| 42 | 7/17/2000 | 1958 | M | 10/8/2010 | IHD, Sudden death |
| 43 | 3/9/2000 | 1932 | F | 4/22/2001 | MS, lower respiratory infection |
| 44 | 1/10/1994 | 1950 | F | 3/15/2010 | MS |
| 45 | 12/20/1993 | 1968 | M | 6/11/2012 | Cause unknown |
| 46^a^ | 2/24/1994 | 1950 | F | 9/1/2010 | Cancer |
| 47 | 4/26/1999 | 1937 | F | 2/14/2011 | MS, PE |
| 48^a^ | 5/4/1994 | 1932 | M | 11/26/2009 | MS, PE, MI |
| 49 | 9/8/1994 | 1931 | M | 4/5/2012 | Cause unknown |
| 50 | 3/7/1995 | 1960 | F | 5/25/1999 | Cardiac arrest |
| 51 | 8/21/1998 | 1946 | F | 11/30/2010 | COPD, diabetes, MS |
| 52 | 4/11/1996 | 1962 | F | 3/28/2011 | MS |
| 53^a^ | 11/10/1998 | 1957 | F | 4/8/2002 | MS, pneumonia |

^a^ prevalent MS case
