# Supplementary material for: Mortality of patients with multiple sclerosis: a cohort study in UK primary care
Source: J Neurol. 2014 May 18;261(8):1508–17. doi: 10.1007/s00415-014-7370-3 (PMC4119255; doi:10.1007/s00415-014-7370-3)
Supplement: Supplementary file 3 — Supplementary material 3 (DOC 100 kb) [file 415_2014_7370_MOESM3_ESM.doc]

**Mortality of Patients with Multiple Sclerosis:
A Cohort Study in UK Primary Care**

SS Jick, L Li, GJ Falcone, ZP Vassilev, M-A Wallander

Corresponding author: Susan Jick DSc, Boston Collaborative Drug Surveillance Program, Boston University School of Public Health, 11 Muzzey Street, Lexington, MA 02421

Telephone: 781-862-6660; Fax: 781-862-1680; email: [sjick@bu.edu](mailto:sjick@bu.edu)

Causes of death among patients diagnosed with MS during the years 2001 to 2006 who subsequently died

| Patient  No. | MS Diagnosis Date | Year of Birth | Sex | Date of Death | Cause of death |
| --- | --- | --- | --- | --- | --- |
| 1 | 11/1/2001 | 1978 | M | 12/9/2008 | Pneumonia, MS, Pressure ulcer |
| 2 | 2/7/2001 | 1929 | F | 12/2/2003 | Pneumonia, MS |
| 3 | 12/22/2001 | 1967 | F | 1/3/2007 | Pneumonia |
| 4^a^ | 5/1/2002 | 1959 | F | 5/9/2005 | Pneumonia |
| 5 | 5/4/2001 | 1916 | F | 12/20/2008 | CVD-tachycardia |
| 6 | 4/16/2003 | 1938 | F | 5/31/2004 | Uterine cancer |
| 7 | 3/14/2005 | 1953 | F | 8/4/2007 | Breast cancer |
| 8 | 11/19/2003 | 1967 | F | 5/1/2009 | Congestive cardiac failure |
| 9 | 5/21/2003 | 1926 | F | 12/11/2005 | Pneumonia, MS |
| 10 | 1/31/2006 | 1954 | M | 1/31/2006 | Alcoholism and MS |
| 11 | 8/23/2005 | 1942 | F | 1/26/2008 | COPD |
| 12 | 9/18/2006 | 1963 | F | 9/17/2007 | MI |
| 13 | 10/17/2001 | 1961 | M | 4/10/2002 | Lung cancer |
| 14 | 9/14/2005 | 1957 | F | 5/3/2006 | Kidney cancer |
| 15 | 9/10/2003 | 1944 | M | 9/25/2006 | Died in hospital - multiple health problems |
| 16 | 7/18/2001 | 1934 | F | 3/12/2002 | pneumonia, CVD, MS |
| 17 | 3/24/2004 | 1958 | F | 2/2/2009 | Breast cancer |
| 18^a^ | 11/27/2003 | 1944 | F | 5/10/2010 | Died in hospital - no cause noted |
| 19 | 2/21/2006 | 1965 | F | 2/1/2009 | No cause noted |
| 20 | 1/8/2003 | 1963 | F | 2/27/2004 | MS |
| 21 | 1/21/2003 | 1928 | F | 10/2/2008 | Pneumonia, MS |
| 22 | 10/18/2004 | 1936 | F | 11/15/2008 | Pneumonia |
| 23 | 1/23/2001 | 1938 | M | 1/8/2008 | MI |
| 24 | 8/21/2002 | 1936 | F | 4/6/2005 | UTI, sepsis, respiratory sx, MS |
| 25 | 4/24/2002 | 1969 | F | 11/6/2007 | Asthma, MS |
| 26^a^ | 7/22/2004 | 1947 | F | 7/31/2008 | Died in hospital - no cause noted |
| 27 | 3/5/2004 | 1963 | F | 2/29/2004 | Bowel obstruction |
| 28 | 4/9/2002 | 1949 | F | 12/27/2002 | MS, respiratory failure, sepsis |
| 29 | 6/17/2002 | 1939 | M | 8/20/2005 | Exacerbation of MS |
| 30 | 3/7/2001 | 1951 | F | 10/15/2003 | No cause noted |
| 31 | 4/19/2001 | 1927 | M | 1/2/2002 | Cerebellar ataxia |
| 32^a^ | 2/8/2005 | 1953 | M | 3/20/2008 | MS |
| 33 | 2/18/2002 | 1965 | F | 1/24/2004 | Pneumonia, MS |
| 34 | 1/1/2001 | 1951 | F | 3/10/2005 | Cancer – lymphoma |
| 35 | 8/5/2003 | 1964 | M | 2/20/2006 | Respiratory disease |
| 36 | 7/25/2003 | 1944 | M | 8/10/2005 | Lung cancer, COPD, IHD |
| 37 | 4/25/2002 | 1942 | M | 1/21/2003 | No cause noted |
| 38 | 12/23/2002 | 1955 | M | 3/12/2006 | Pneumonia, MS |
| 39 | 11/15/2002 | 1956 | F | 1/24/2005 | Pneumonia, respiratory arrest, MS |
| 40 | 8/7/2001 | 1952 | F | 12/13/2004 | MI |
| 41 | 11/17/2006 | 1938 | F | 2/9/2009 | Cause unknown |
| 42 | 8/1/2001 | 1947 | F | 3/6/2003 | Cause unknown |
| 43^a^ | 2/1/2003 | 1989 | F | 7/20/2007 | Cause unknown |
| 44 | 7/8/2004 | 1932 | F | 10/23/2005 | Cancer, anemia |
| 45^a^ | 8/25/2004 | 1929 | M | 11/3/2011 | MS, pneumonia |
| 46 | 12/6/2002 | 1939 | M | 7/27/2004 | MS, pneumonitis |
| 47 | 2/6/2003 | 1969 | M | 11/9/2009 | MS, pneumonia |
| 48^a^ | 12/9/2002 | 1946 | F | 7/25/2009 | Cancer |
| 49 | 11/28/2001 | 1922 | F | 7/25/2007 | MS, UTI |
| 50 | 7/9/2003 | 1922 | F | 5/27/2010 | Cellulitis |
| 51 | 3/5/2004 | 1935 | F | 1/30/2010 | Chest infection |
| 52 | 9/13/2005 | 1977 | F | 5/13/2012 | Cause unknown |
| 53 | 6/1/2005 | 1963 | F | 9/30/2011 | Cause unknown |
| 54 | 7/8/2003 | 1947 | F | 7/27/2004 | Motor neurone disease, bronchopneumonia |
| 55 | 5/8/2001 | 1959 | F | 3/17/2010 | MS, pneumonia |
| 56 | 7/26/2004 | 1944 | M | 10/15/2011 | Cancer |
| 57 | 3/14/2003 | 1927 | F | 5/30/2011 | Atherosclerosis |
| 58 | 4/29/2003 | 1955 | F | 1/8/2010 | MS, pneumonia |
| 59 | 5/18/2005 | 1950 | M | 2/4/2009 | Motor neurone disease |
| 60 | 6/1/2003 | 1975 | M | 2/24/2011 | MS, pneumonia |
| 61 | 8/3/2004 | 1975 | F | 9/22/2007 | MS |
| 62 | 10/1/2001 | 1954 | M | 9/28/2010 | Cancer |
| 63 | 9/7/2001 | 1949 | F | 1/28/2007 | Cancer |
| 64 | 3/11/2004 | 1959 | F | 4/19/2010 | Pneumonia |
| 65 | 1/7/2002 | 1927 | M | 3/17/2012 | MS, pneumonia |
| 66 | 1/1/2002 | 1954 | F | 2/6/2012 | Cause unknown |
| 67 | 6/5/2003 | 1948 | M | 1/17/2007 | MS |
| 68 | 7/1/2003 | 1949 | F | 3/10/2010 | Respiratory arrest |
| 69^a^ | 4/24/2003 | 1965 | M | 8/25/2011 | MS, pneumonia |
| 70 | 11/7/2001 | 1949 | F | 1/3/2011 | Cancer |
| 71 | 2/24/2003 | 1949 | F | 8/20/2010 | Cause unknown |
| 72 | 7/19/2002 | 1944 | F | 6/6/2005 | MS, PE, pancreatitis |
| 73 | 1/1/2002 | 1965 | F | 4/11/2011 | MS |
| 74 | 3/4/2002 | 1940 | M | 3/20/2011 | Stroke, MS, IHD, diabetes |
| 75 | 7/10/2003 | 1968 | F | 11/22/2007 | MS, VTE |
| 76 | 6/13/2006 | 1917 | F | 11/16/2010 | MS, pneumonia |
| 77 | 5/22/2006 | 1955 | F | 11/6/2009 | Cause unknown |

^a^ prevalent MS case
