# Supplementary material for: Mortality of patients with multiple sclerosis: a cohort study in UK primary care
Source: J Neurol. 2014 May 18;261(8):1508–17. doi: 10.1007/s00415-014-7370-3 (PMC4119255; doi:10.1007/s00415-014-7370-3)
Supplement: Supplementary file 4 — Supplementary material 4 (DOC 33 kb) [file 415_2014_7370_MOESM4_ESM.doc]

**Mortality of Patients with Multiple Sclerosis:
A Cohort Study in UK Primary Care**

SS Jick, L Li, GJ Falcone, ZP Vassilev, M-A Wallander

Corresponding author: Susan Jick DSc, Boston Collaborative Drug Surveillance Program, Boston University School of Public Health, 11 Muzzey Street, Lexington, MA 02421

Telephone: 781-862-6660; Fax: 781-862-1680; email: [sjick@bu.edu](mailto:sjick@bu.edu)

Basic characteristics of definite or probable MS cases and matched referent subjects at cohort entry

| **Characteristic** | **MS cases**  **N = 1507**  **(n [%])** | **Referents**  **N = 15070**  **(n [%])** |
| --- | --- | --- |
| **Mean age (years) at cohort entry (index date; SD)** | 41.59 (11.40) | 41.53 (11.40) |
| **Sex**  Males  Females | 397 (26.34)  1110 (73.66) | 3970 (26.34)  11100 (73.66) |
| **Smoking status**  Current^a^  Former  Never  Unknown | 463 (30.72)  199 (13.21)  648 (43.00)  197 (13.07) | 3648 (24.21)  1942 (12.89)  7389 (49.03)  2091 (13.88) |
| **BMI (kg/m^2^)**  < 18.5  18.5–24.99  25.0–29.99  ≥ 30  Unknown | 35 (2.32)  583 (38.69)  361 (23.95)  205 (13.60)  323 (21.43) | 312 (2.07)  5788 (38.41)  3388 (22.48)  1971 (13.08)  3611 (23.96) |
| **Alcohol abuse^a^** | 17 (1.13) | 295 (1.96) |
| **Mean length of recorded medical history (years; SD)**  Before index date  Follow-up after index date | 7.86 (4.42)  8.18 (4.41) | 7.97 (4.41)  8.05 (4.47) |

^a^P < 0.05 for comparison between patients with MS and matched referent subjects.

BMI, body mass index; MS, multiple sclerosis; SD, standard deviation
