# Supplementary material for: Mortality of patients with multiple sclerosis: a cohort study in UK primary care
Source: J Neurol. 2014 May 18;261(8):1508–17. doi: 10.1007/s00415-014-7370-3 (PMC4119255; doi:10.1007/s00415-014-7370-3)
Supplement: Supplementary file 5 — Supplementary material 5 (DOC 31 kb) [file 415_2014_7370_MOESM5_ESM.doc]

**Mortality of Patients with Multiple Sclerosis:
A Cohort Study in UK Primary Care**

SS Jick, L Li, GJ Falcone, ZP Vassilev, M-A Wallander

Corresponding author: Susan Jick DSc, Boston Collaborative Drug Surveillance Program, Boston University School of Public Health, 11 Muzzey Street, Lexington, MA 02421

Telephone: 781-862-6660; Fax: 781-862-1680; email: [sjick@bu.edu](mailto:sjick@bu.edu)

Comorbidities of definite or probable MS cases and matched referent subjects at cohort entry

| **Characteristic** | **MS cases**  **N = 1507**  **(n [%])** | **Referents**  **N = 15070**  **(n [%])** |
| --- | --- | --- |
| **Chronic comorbidities^a^**  COPD and asthma  Depression^b^  Diabetes  Hypertension  Heart disease  Cancer | 247 (16.39)  415 (27.54)  26 (1.73)  104 (6.90)  24 (1.59)  39 (2.59) | 2407 (15.97)  3063 (20.33)  309 (2.05)  1202 (7.98)  341 (2.26)  431 (2.86) |
| **Acute comorbidities^c^**  Acute respiratory infection  Pneumonia and influenza  Urinary tract infection^b^  Skin infection  Eye or Ear infection  Other infection  Dyspepsia | 210 (13.93)  16 (1.06)  82 (5.44)  133 (8.83)  4 (0.27)  122 (8.10)  26 (1.73) | 2231 (14.80)  151 (1.00)  569 (3.78)  1285 (8.53)  27 (0.18)  1053 (6.99)  230 (1.53) |
| **Charlson Comorbidity Index at cohort entry**  Low (0)  Medium (1–2)  High (> 2) | 1166 (77.37)  323 (21.43)  18 (1.19) | 11794 (78.26)  3122 (20.72)  154 (1.02) |

^a^Ever before, or at cohort entry.

^b^P < 0.05 for comparison between patients with MS and matched referent subjects.

^c^During the year before, or at index date.

COPD, chronic obstructive pulmonary disorder; MS, multiple sclerosis
