# Supplementary material for: Mortality of patients with multiple sclerosis: a cohort study in UK primary care
Source: J Neurol. 2014 May 18;261(8):1508–17. doi: 10.1007/s00415-014-7370-3 (PMC4119255; doi:10.1007/s00415-014-7370-3)
Supplement: Supplementary file 6 — Supplementary material 6 (DOC 30 kb) [file 415_2014_7370_MOESM6_ESM.doc]

**Mortality of Patients with Multiple Sclerosis:
A Cohort Study in UK Primary Care**

SS Jick, L Li, GJ Falcone, ZP Vassilev, M-A Wallander

Corresponding author: Susan Jick DSc, Boston Collaborative Drug Surveillance Program, Boston University School of Public Health, 11 Muzzey Street, Lexington, MA 02421

Telephone: 781-862-6660; Fax: 781-862-1680; email: [sjick@bu.edu](mailto:sjick@bu.edu)

Comedications of definite or probable MS cases and matched referent subjects at cohort entry

| **Characteristic** | **All MS cases**  **N = 1507**  **(n [%])** | **Referents**  **N = 15070**  **(n [%])** |
| --- | --- | --- |
| **Comedications (at cohort entry [index date] or within the 6 months before)**  Systemic glucocorticoids^a^  Antidepressants^a^  Anticonvulsants^a^  Antidiabetics  Opioids^a^  NSAIDs^a^  Statins  Antibiotics  Muscle relaxants^a^  Antipsychotics^a^  Anti-Parkinson drugs^a^  PPIs | 154 (10.22)  301 (19.97)  102 (6.77)  18 (1.19)  271 (17.98)  260 (17.25)  39 (2.59)  328 (21.77)  136 (9.02)  97 (6.44)  20 (1.33)  65 (4.31) | 573 (3.80)  1303 (8.65)  189 (1.25)  223 (1.48)  1279 (8.49)  1466 (9.73)  337 (2.24)  2972 (19.72)  319 (2.12)  326 (2.16)  39 (0.26)  524 (3.48) |

^a^P < 0.05 for comparison between patients with MS and matched referent subjects.

MS, multiple sclerosis; NSAIDs, non-steroidal anti-inflammatory drugs; PPIs, proton pump inhibitors
