# Supplementary material for: Mortality of patients with multiple sclerosis: a cohort study in UK primary care
Source: J Neurol. 2014 May 18;261(8):1508–17. doi: 10.1007/s00415-014-7370-3 (PMC4119255; doi:10.1007/s00415-014-7370-3)
Supplement: Supplementary file 7 — Supplementary material 7 (DOC 38 kb) [file 415_2014_7370_MOESM7_ESM.doc]

**Mortality of Patients with Multiple Sclerosis:
A Cohort Study in UK Primary Care**

SS Jick, L Li, GJ Falcone, ZP Vassilev, M-A Wallander

Corresponding author: Susan Jick DSc, Boston Collaborative Drug Surveillance Program, Boston University School of Public Health, 11 Muzzey Street, Lexington, MA 02421

Telephone: 781-862-6660; Fax: 781-862-1680; email: [sjick@bu.edu](mailto:sjick@bu.edu)

Death rate per 1000 person-years in patients with definite or probable MS and matched referent subjects overall, stratified by age at first diagnosis of MS and sex

|  | **Deaths (N)** | **Population size** | **Person-years** | **Death rates (95% CI)** |
| --- | --- | --- | --- | --- |
| **Overall**  Patients with MS  Referent Subjects | 101  438 | 1507  15070 | 12326  121357 | 8.19 (6.71–9.91)  3.61 (3.28–3.96) |
| **Age group at diagnosis (years)** | | | | |
| **< 30**  Patients with MS  Referent Subjects | 6  4 | 223  2249 | 1792  16134 | 3.35 (1.36–6.96)  0.25 (0.08–0.60) |
| **30–39**  Patients with MS  Referent Subjects | 18  43 | 445  4452 | 3598  35099 | 5.00 (3.06–7.75)  1.23 (0.90–1.64) |
| **40–49**  Patients with MS  Referent Subjects | 30  90 | 473  4726 | 3980  40207 | 7.54 (5.18–10.62)  2.24 (1.81–2.74) |
| **50–59**  Patients with MS  Referent Subjects | 24  152 | 274  2734 | 2297  22802 | 10.45 (6.85–15.31)  6.67 (5.67–7.79) |
| **≥ 60**  Patients with MS  Referent Subjects | 23  149 | 92  909 | 658  7115 | 34.95 (22.70–51.62)  20.94 (17.78–24.51) |
| **Sex** | | | | |
| **Male**  Patients with MS  Referent Subjects | 33  182 | 397  3970 | 3242  31697 | 10.18 (7.12–14.13)  5.74 (4.95–6.62) |
| **Female**  Patients with MS  Referent Subjects | 68  256 | 1110  11100 | 9084  89660 | 7.49 (5.86–9.43)  2.86 (2.52–3.22) |

CI, confidence interval; MS, multiple sclerosis
