# Supplementary material for: Mortality of patients with multiple sclerosis: a cohort study in UK primary care
Source: J Neurol. 2014 May 18;261(8):1508–17. doi: 10.1007/s00415-014-7370-3 (PMC4119255; doi:10.1007/s00415-014-7370-3)
Supplement: Supplementary file 8 — Supplementary material 8 (DOC 34 kb) [file 415_2014_7370_MOESM8_ESM.doc]

**Mortality of Patients with Multiple Sclerosis:
A Cohort Study in UK Primary Care**

SS Jick, L Li, GJ Falcone, ZP Vassilev, M-A Wallander

Corresponding author: Susan Jick DSc, Boston Collaborative Drug Surveillance Program, Boston University School of Public Health, 11 Muzzey Street, Lexington, MA 02421

Telephone: 781-862-6660; Fax: 781-862-1680; email: [sjick@bu.edu](mailto:sjick@bu.edu)

Hazard ratios and 95% confidence intervals for all-cause mortality among definite or probable MS cases versus matched referent subjects.

| **Characteristic** | **Death in MS patients**  **N = 101**  **n [%]** | **Deaths in non-MS referents**  **N = 438**  **n [%]** | **HR (95% CI)** | |
| --- | --- | --- | --- | --- |
|  |  |  | **Model 1^a^** | **Model 2^b^** |
| **Overall** | 101 (100) | 438 (100) | 2.27 (1.83–2.81) | 1.72 (1.38–2.15) |
| **Age at first MS diagnosis (years)** | | | | |
| < 30  30–39  40–49  50–59  ≥ 60 | 6 (5.94)  18 (17.82)  30 (29.70)  24 (23.76)  23 (22.77) | 4 (0.91)  43 (9.82)  90 (20.55)  152 (34.70)  149 (34.02) | 12.83 (3.60–45.72)  4.12 (2.38–7.14)  3.39 (2.25–5.13)  1.55 (1.01–2.38)  1.67 (1.08–2.59) | 12.37 (3.38–45.30)  3.20 (1.83–5.62)  2.50 (1.62–3.86)  1.25 (0.80–1.97)  1.48 (0.94–2.32) |
| **Sex** | | | | |
| Male  Female | 33 (32.67)  68 (67.33) | 182 (41.55)  256 (58.45) | 1.75 (1.21–2.54)  2.63 (2.01–3.43) | 1.31 (0.89–1.91)  1.96 (1.48–2.58) |

^a^Adjusted for matching variables (age, sex, index date, GP, and length of medical history recorded before the index date).

^b^Adjusted for smoking and comedications (including antidepressants, opioids, muscle relaxants) within 6 months before or at the index date, in addition to the matching variables.

CI, confidence interval; HR, hazard ratio; MS, multiple sclerosis
